# Evaluation, prevention, and treatment of inferior alveolar nerve injury in bilateral sagittal split mandibular osteotomy

**DOI:** 10.1186/s40902-025-00486-5

**Published:** 2025-10-10

**Authors:** Hao-ran Zhao, Ning Zhao, Yao-xiang Xu, Fu-chen Wang, Wen-lin Xiao

**Affiliations:** The Affiliated Hospital of Qingdao University, Qingdao City, China

**Keywords:** Bilateral sagittal split osteotomy, Inferior alveolar nerve, Neurosensory disturbance, Preoperative imaging, Surgical instrumentation

## Abstract

**Background:**

Bilateral sagittal split osteotomy (BSSO) is a widely adopted surgical procedure for correcting mandibular deformities, yet neurosensory disturbance (NSD) of the inferior alveolar nerve (IAN) remains a significant postoperative complication. This complication adversely impacts patients' quality of life due to persistent sensory abnormalities in the lower lip and chin region.

**Main body:**

This narrative review summarizes anatomical risks and prevention/management strategies. Cone-beam CT (CBCT) may clarify canal anatomy and support risk stratification. Nerve-sparing osteotomy modifications are intended to limit traction and direct exposure. Fixation choice may influence surrogate and early clinical outcomes; monocortical miniplates (MCF) may be associated with lower radiographic canal penetration and earlier recovery than bicortical screws (BCF), whereas long-term clinical differences are uncertain. Piezoelectric/ultrasonic devices may improve precision and reduce tissue trauma, and virtual planning with 3D-printed guides may support safer osteotomy paths. For established IAN injury, photobiomodulation (PBM) may support earlier recovery; corticosteroid effects are route-dependent—intravenous dexamethasone mainly reduces edema with uncertain NSD benefit, while local betamethasone at closure may yield early improvement.

**Conclusion:**

Comprehensive management likely requires integrated preoperative assessment, refined technique, and postoperative adjuncts. Current evidence supports considering multimodal approaches—imaging-guided planning, nerve-sparing modifications, and PBM—to potentially reduce NSD and enhance early recovery, while standardized protocols and larger studies are needed before firm recommendations.

## Introduction

Bilateral sagittal split osteotomy is a prevalent surgical technique in orthognathic surgery aimed at correcting mandibular deformities. Nevertheless, the close proximity of the inferior alveolar nerve during the procedure frequently results in neurosensory disturbance, which manifests as abnormal sensations in the lower lip and chin, thereby negatively impacting postoperative quality of life [1]. The reported incidence of NSD varies significantly across studies, with figures ranging from 9% to 84.6% [2], and its severity and recovery trajectory are influenced by temporal factors. A prospective study conducted by Hanfesh et al. revealed that tactile and pinprick sensory deficits were highly prevalent at 1 week postoperatively, with some patients continuing to exhibit residual abnormalities at 3 months [3]. Hanzelka et al. found that nearly 50% of patients experienced sensory abnormalities at 4 weeks, although the incidence decreased to 3% at 1 year [4]. Furthermore, the selection of assessment methods may affect the interpretation of outcomes. Colella et al. conducted a systematic review revealing discrepancies between objective assessments and subjective questionnaires in evaluating nerve injury rates at 7 days postoperatively [1]. This suggests that patients’ subjective perceptions may not accurately reflect actual nerve functional recovery. While the majority of studies indicate that nerve function is reversible, Fridrich et al. identified no statistically significant differences in sensory test outcomes between preoperative and 6-month postoperative evaluations [5]. Nonetheless, a subset of patients remains susceptible to long-term or permanent injury. Shibata et al. reported a 13.1% incidence of NSD at 1 year [6], whereas multicenter studies by van Merkesteyn et al. [7] and Mensink et al. [8] documented rates of 8.3% and 8.9%, respectively. Long-term follow-up data demonstrate heterogeneous recovery patterns. A retrospective study by Nesari et al. found that 24% of patients continued to experience sensory disturbances 2.5 years postoperatively [9]. A systematic review highlighted that the occurrence of NSD is influenced by multiple factors, including surgical technique, precision of nerve localization, and individual variability [10]. In conclusion, the effective management of IAN injury following BSSO necessitates the identification of risk factors, the implementation of precise surgical techniques for prevention, and the application of post-injury intervention strategies aimed at reducing complication rates [10]. This review consolidates existing evidence to enhance surgical protocols and improve patient outcomes.

### Anatomical factors influencing IAN-related NSD after BSSO

Methods for evaluating IAN injury are classified into subjective, semi-objective, and objective categories [11]. The selection of an assessment method plays a crucial role in determining the reliability and clinical relevance of the outcomes. Subjective evaluations predominantly depend on patient-reported sensory changes in the lower lip and chin, typically gathered through questionnaires. Although easy to administer, this approach is vulnerable to individual perceptual variability [12, 13]. For example, research by Al-Bishri et al. indicates that patients often report a higher sensitivity to sensory abnormalities than the actual extent of nerve functional recovery [12]. Semi-objective assessments encompass tests such as static light touch and directional discrimination. Teerijoki-Oksa et al. demonstrated that tactile detection threshold (TDT) testing offers greater sensitivity for detecting intraoperative nerve injury compared to brush-stroke direction (BSD) or warm/cold discrimination (W/C) tests [14]. Nonetheless, these methods are susceptible to variability due to inadequate standardization and issues with patient compliance [11]. Objective evaluations, particularly electrophysiological techniques, significantly enhance diagnostic accuracy. Nerve conduction studies (NCS) are instrumental in detecting damage to thickly myelinated Aβ fibers [14], whereas quantitative sensory testing (QST) is employed to assess thin fiber function. However, these methodologies are constrained by substantial equipment costs and operational complexity [11]. A systematic review conducted by Colella et al. highlights that objective methods, such as trigeminal somatosensory evoked potentials (TSEP), exhibit high sensitivity within the initial 3 months postoperatively [1]. Nonetheless, central compensatory mechanisms may obscure peripheral nerve damage over time [14]. Current research is hindered by challenges, including inconsistent assessment criteria and limited sample sizes, with most electrophysiological studies comprising fewer than 30 cases [11, 14], thereby restricting generalizability. Future research endeavors should focus on fostering multicenter collaborations to establish standardized protocols, investigating the combined application of subjective and objective methods (e.g., integrating questionnaires with QST), increasing sample sizes, and elucidating optimal testing strategies for both early postoperative monitoring and long-term follow-up. These measures aim to improve diagnostic accuracy for IAN injury after BSSO and refine targeted therapeutic approaches [1, 11] (Table 1).

**Table 1:** Classification of IAN injury assessment methods

Category	Example tests	Key advantages	Major limitations
Subjective	VAS numbness scale Patient symptom diaries	Clinically efficient Reflects QoL impact	Higher false-positive risk with purely subjective tools
Semi-objective	Brush-stroke direction (BSD) Static light touch (SLT)	Low-cost equipment Bedside applicability	Low sensitivity for partial injury
Objective	Nerve conduction studies (NCS) Quantitative sensory testing (QST)	Quantifies fiber-specific damage Detects subclinical injury	Requires specialized equipment Limited clinical access

This table provides a qualitative comparison of IAN sensory assessment methods and does not present numeric endpoints. Evidence that (i) purely subjective scales may over-call early abnormalities, (ii) semi-objective bedside tests have limited sensitivity for partial injuries, and (iii) objective methods (e.g., TSEP/NCS/QST) tend to be more sensitive within ≤3 months post-op but are constrained by availability is summarized in Refs. [1, 11, 12, 14]. If any numeric claims are added to table cells, please list the exact value and its reference here

### Methods to avoid or reduce IAN injury after BSSO

#### Preoperative imaging assessment

Accurate preoperative imaging is essential for reducing the risk of IAN injury following BSSO and for enhancing surgical planning. CBCT with its three-dimensional imaging capabilities effectively delineates the spatial relationship between the mandibular canal and surrounding structures, as well as identifies areas of cortical bone thinning, thereby informing the planning of the osteotomy path [15, 16]. Ylikontiola et al. have confirmed that CBCT significantly improves the localization of the mandibular canal and the detection of high-risk anatomical variations [17]. Furthermore, a study by Kanneth et al., which included 30 patients, demonstrated that CBCT-based preoperative evaluation significantly reduces the incidence of NSD [18]. A randomized clinical trial conducted by Hassani et al. indicated that the integration of CBCT with conventional imaging techniques enhances the surgeon’s comprehension of the three-dimensional positioning of the mandibular canal, thereby facilitating more precise nerve preservation and supporting postoperative functional recovery [19].

Panoramic radiography (orthopantomogram, OPG), while cost-effective and widely accessible, is associated with certain limitations. As evidenced by Politis et al., OPG demonstrates a higher detection rate of the mandibular canal in the retromolar and mandibular angle regions; however, its efficacy in visualizing the area near the mental foramen is inadequate [20]. Postoperatively, the visibility of the mandibular canal significantly diminishes in the short term, necessitating a recovery period of approximately 12 months to return to preoperative visibility levels. Furthermore, OPG is constrained in its ability to accurately assess the distance between the mandibular canal and the buccal bone cortex, as well as in identifying complex anatomical variations. Despite these limitations, OPG remains a valuable adjunctive tool for preliminary evaluations and postoperative monitoring [20]. In contrast, computed tomography (CT) is highly effective in localizing the mandibular canal. Research indicates that CT outperforms both conventional spiral tomography and panoramic radiography in visualizing the buccolingual position of the mandibular canal [21]. The integrated utilization of CBCT and OPG capitalizes on their complementary advantages: CBCT provides high-resolution three-dimensional imaging essential for precise preoperative planning, while OPG facilitates rapid screening and postoperative monitoring. This multimodal approach reduces intraoperative risks associated with nerve traction or transection through comprehensive multidimensional assessment [17, 19]. By strategically combining these imaging modalities and leveraging the surgeon’s expertise in identifying anatomical variations, critical technical support is furnished for nerve protection during BSSO. Such strategies effectively diminish postoperative IAN injury and improve clinical outcomes.

#### Surgical technique modifications

To mitigate the risk of IAN injury following BSSO, various modified surgical techniques have been proposed and evaluated. These techniques primarily focus on optimizing osteotomy pathways, minimizing nerve traction, and reducing intraoperative nerve exposure.

Chortrakarnkij et al. advanced the Obwegeser-Dal Pont technique by minimizing medial periosteal stripping and optimizing the positioning of the osteotomy line, resulting in a 3.5% incidence of IAN injury at 12 months postoperatively [22]. Schlund et al. introduced a technique preserving the inferior border, which obviated the need for nerve decompression and reported a 12.5% rate of hypoesthesia at a 1-year follow-up, while effectively mitigating mechanical stress on the IAN during surgery [23].

Kumaran et al. incorporated an additional mandibular inferior border osteotomy to ensure the complete containment of the IAN within the distal bone segment, leading to full sensory recovery for all patients within 3 months postoperatively [24]. Schoen et al. through experimental validation using porcine mandible models demonstrated that modified osteotomy techniques, including the inferior border osteotomy, resulted in a 30% reduction in splitting force [25]. Notably, 75% of specimens exhibited fracture patterns along the inferior border rather than disruption of the mandibular canal, thereby confirming the neuroprotective efficacy of these modifications.

The high oblique sagittal split osteotomy (HSSO) positions the osteotomy line 3 mm superior to the entry point of the IAN [26]. In a cohort of 50 patients, this technique resulted in the absence of postoperative sensory abnormalities, with pinprick test outcomes at 6 months post-surgery being indistinguishable from preoperative baselines. This indicates that high-level osteotomy effectively avoids direct involvement of the nerve trunk. Parameswaran and Panneerselvam introduced the parallel osteotomy splitting technique (POST), which mitigates mechanical stress on the IAN and results in significantly lower rates of NSD at 3 to 12 months postoperatively compared to conventional methods [27]. Politis et al. modified the buccal osteotomy technique by preserving the lingual bone plate, thereby reducing IAN injury rates from 36.4% to 9.73% [20]. Hunsuck further minimized fracture risks and direct nerve exposure by optimizing the lingual osteotomy pathway [28]. While these techniques demonstrate promising short-term outcomes, as noted by Kumaran et al. [24], Schlund et al. underscore the necessity for extended follow-up studies to confirm their long-term stability [23].

The integration of anatomically guided optimization of osteotomy pathways (e.g., the superior positioning of horizontal split sagittal osteotomies) and strategies for controlled nerve exposure (e.g., preservation of the buccal bone plate) collectively contribute to mitigating the risk of IAN injury during BSSO. Nevertheless, the criteria for selecting specific techniques and their synergistic applications warrant further investigation to achieve a balance between therapeutic efficacy and procedural complexity.

#### Selection of internal fixation techniques

The choice of internal fixation technique in BSSO plays a crucial role in influencing the risk of IAN injury, with BCF and MCF being the primary options under scrutiny.

Numerous studies have indicated that BCF may be associated with a heightened risk of postoperative IAN injury. Fujioka et al. observed delayed neural recovery in patients treated with BCF [29]. A long-term comparative study by Yamashita et al. [30] demonstrated that the MCF group experienced a 30% faster sensory recovery compared to the BCF group. Furthermore, in a rhesus macaque model undergoing bilateral BSSO, Hu et al. demonstrated that MCF fixation, compared to BCF on the contralateral sides, facilitated faster neural recovery [31]. Sinha et al. employed CBCT and identified a significantly higher incidence of nerve canal penetration in BCF cases (58.8%) compared to MCF cases (6%) (odds ratio [OR] = 52.5), highlighting a clear radiographic advantage for MCF [32].

Although Yamashita et al. did not observe a statistically significant difference in NSD rates between the two groups at a 5-year follow-up, the MCF group demonstrated advantages in the pace of early functional recovery [30]. Tabrizi et al., employing VAS assessments, demonstrated that patients with MCF fixation exhibited superior neurosensory recovery at 1-year post-BSSO compared to those receiving alternative fixation techniques [33]. A retrospective analysis by Yeo et al. indicated that miniplate fixation might be associated with slightly higher long-term NSD rates, although the difference was not statistically significant due to the limited sample size [34].

To achieve a balance between stability and neuroprotection, hybrid fixation techniques that combine BCF and MCF have been proposed. Pereira et al. presented preliminary evidence suggesting that these techniques enhance segmental stability while mitigating the risks associated with IAN injury, although the long-term outcomes have yet to be validated [35].

Current evidence suggests that MCF may be a preferable approach for cases with a high risk to the IAN, as it is associated with lower rates of radiographically detected canal penetration and may support faster initial recovery. However, the choice of fixation should be individualized, considering that BCF may still be indicated in cases requiring exceptional stability. In contrast, BCF should be reserved for complex cases that necessitate exceptional stability, provided that intraoperative navigation or CBCT-guided planning is utilized to prevent penetration of the nerve canal [32].

Despite accumulating evidence suggesting that MCF may reduce the risk of radiographically evident nerve injury and facilitate faster early sensory recovery, the optimal choice of fixation remains an area of discussion. Some studies reported a trend towards slightly higher long-term NSD rates with miniplate fixation (MCF), although the difference lacked statistical significance, potentially due to sample size limitations [34]. Furthermore, BCF continues to be advocated by some surgeons for complex cases requiring exceptional stability (e.g., large mandibular setbacks), provided meticulous intraoperative navigation or CBCT-guided planning is employed to avoid nerve canal penetration [32]. The long-term outcomes and neuroprotective efficacy of hybrid fixation techniques also warrant further validation through larger studies [35].

#### Modification of surgical instruments

The choice of surgical instruments in BSSO plays a crucial role in influencing the risk of intraoperative nerve injury and postoperative recovery outcomes. Comparative studies of traditional versus novel instruments offer valuable insights for optimizing instrument selection. Specifically, research by Verweij et al. [36] and Mensink et al. [37] demonstrated that replacing traditional chisels with osteotomes and separators allows for more controlled creation of the lingual fracture lines without increasing IAN injury risks.

Traditional osteotomy instruments are known to generate both vibration and thermal energy, which may elevate the risk of neuropathic complications. In an effort to address these concerns, Raffaini et al. introduced a hybrid technique that integrates oscillating saws with piezoelectric devices, using a porcine mandible model [38]. Their findings indicated that this hybrid approach enhanced the precision of osteotomies, reduced operative time, decreased lingual fracture rates by 20%, and minimized neural trauma. Conversely, in vitro studies conducted by Cagri Gencer et al. found no significant differences between conventional chisel-mallet techniques and magnetic mallet-chisel systems in terms of lingual fracture patterns, osteotomy duration, or incidence of neural injury [39].

Piezoelectric devices, which utilize micro-vibrations, allow for selective bone cutting. A retrospective analysis by D’Agostino et al. revealed that 83% of patients treated with piezoelectric devices experienced only mild sensory alterations during postoperative neurological examinations [40]. Furthermore, research by Koba et al. demonstrated that piezoelectric osteotomy reduced operative time by 30% compared to traditional methods, with postoperative neural paralysis rates declining to 8% at 3 months, compared to 24% in the conventional group [41]. Sobol et al. conducted a prospective split-mouth study, reporting that the group utilizing piezoelectric saws exhibited superior final neural sensory scores and a 15% enhancement in two-point discrimination recovery rates, although the timelines for functional sensory recovery were comparable to those of traditional instruments [42]. In contrast, Köhnke et al. advised caution, noting that piezoelectric technology does not unequivocally prevent sensory deficits, and highlighted the importance of considering operator expertise and case-specific factors in its application [43].

The efficacy of ultrasonic osteotomes has been corroborated by several studies. A retrospective cohort study by Kokuryo et al. indicated a 42% reduction in the incidence of NSD at 3 months postoperatively in the ultrasonic group compared to the conventional group, with increased effectiveness observed when nerves were closely adherent to the buccal cortical bone [44]. Ruiz Valero et al. found that the ultrasonic osteotome group achieved sensory recovery within 4.2 weeks, significantly shorter than the 6.8 weeks observed in the traditional group [45]. Dammous et al. have further substantiated, through three-dimensional computed tomography, that ultrasonic osteotomes enhance the morphology of lingual fracture lines and mitigate complications associated with pterygomaxillary separation [46].

Nonetheless, the implementation of these novel instruments requires precise technique. Piezoelectric devices necessitate stringent control over cutting depth to prevent inadvertent neural damage [10], whereas ultrasonic osteotomes require adjustments in frequency and amplitude to achieve a balance between cutting efficiency and soft tissue preservation [44]. Raffaini et al. also highlighted the critical importance of gentle intraoperative maneuvers and the careful blunt dissection of nerves [38].

While the aforementioned studies suggest benefits of piezoelectric devices in reducing neural trauma and operative time, their superiority over conventional instruments in consistently preventing neurosensory disturbance is not universally accepted. Köhnke et al. advised caution, noting that piezoelectric osteotomy did not unequivocally prevent sensory deficits in their investigation [43]. This highlights that the effectiveness of these advanced instruments may be influenced by operator expertise and case-specific anatomical factors. The significant cost associated with piezoelectric technology and the learning curve required for its proficient use also represent practical considerations impacting its widespread adoption.

Current studies are constrained by limited sample sizes, such as the study by D’Agostino et al. [40], which included only 52 cases, and by methodological heterogeneity. Future research should focus on multicenter, large-scale studies to elucidate the optimal indications for various instruments and investigate real-time intraoperative monitoring technologies to enhance the safety of BSSO [10, 44].

#### Application of digital guides and virtual design

Recent advancements in digital technology have introduced innovative approaches for safeguarding the IAN during BSSO. The integration of VSP, three-dimensional printed surgical guides, and intraoperative imaging has facilitated enhanced precision in surgical procedures.

Preoperative utilization of three-dimensional modeling software, such as Dolphin Imaging and Mimics, enables the reconstruction of the mandible and IAN pathways, thereby optimizing the design of osteotomy lines to circumvent the neurovascular canal. Grillo et al. employed Blender software to design a surgical guide that provided accurate osteotomy guidance intraoperatively [47]. Postoperative computed tomography scans verified that the osteotomy lines were in close proximity to the neurovascular canal without inflicting injury, while the operative time was reduced by 20%. Furthermore, Chen et al. refined the sagittal split trajectory by extending the osteotomy line 5 mm posterior to the mental foramen, which minimized bony gaps and achieved a 100% bone contact rate, thus mitigating the risk of neural compression [48].

Intraoperative imaging technologies have markedly enhanced surgical safety. Agbaje et al. employed the Artis Zeego system for real-time CT scanning, documenting transient IAN injury in 80% of patients at 6 weeks postoperatively, with all patients achieving complete recovery after 1 year [49]. This finding highlights the critical role of intraoperative imaging adjustments in averting permanent neural damage. Abdel-Moniem Barakat et al. utilized Mimics software for three-dimensional annotation of the IAN pathway on CBCT scans, thereby dynamically guiding the osteotomy direction to minimize mechanical traction or accidental transection of the IAN [50]. Furthermore, their modified osteotomy technique for managing the inferior border division reduced the risk of nerve attachment to the proximal bone segment [49].

Computer-assisted surgical simulation has significantly improved biomechanical efficiency in surgical procedures. Chen et al. introduced a novel curved osteotomy design that achieved a 100% bone contact rate, markedly surpassing traditional methods and eliminating the necessity for bone grafting [48]. This innovative approach resulted in a 35% reduction in the incidence of postoperative NSD. The development of customized surgical guides represents a crucial advancement in this field. Grillo et al. demonstrated the efficacy of 3D-printed guides in accurately translating virtual surgical plans into intraoperative execution, thereby minimizing human error [47]. Similarly, Abdel-Moniem Barakat et al. developed bone-supported guides designed to maintain condylar positioning, effectively reducing the risk of nerve traction [50]. These digital technologies offer significant advantages in terms of preoperative planning accuracy, real-time intraoperative feedback, and postoperative bone segment stability.

Future research should focus on developing user-friendly surgical simulation software integrated with AI-driven automated planning modules, advancing portable CBCT-based intraoperative imaging systems, and refining guide designs using biomechanical models to further mitigate the risk of IAN injury during BSSO [47, 50].

### Management of IAN injury following BSSO

#### Photobiomodulation therapy

Photobiomodulation, a non-invasive therapeutic approach, facilitates the recovery of the IAN following BSSO through a range of biological mechanisms. These mechanisms include the activation of mitochondrial cytochrome c oxidase, which enhances the efficiency of ATP synthesis [51], the suppression of inflammatory mediator release [52], and the stimulation of Schwann cell proliferation and nerve growth factor secretion [53].

Clinical studies indicate that the efficacy of PBM is time-dependent. Suboptimal outcomes are noted within the first 48 h postoperatively [54], whereas prolonged treatment over a period of 30 days significantly improves two-point discrimination and visual analogue scale (VAS) pain scores [55, 56]. Recovery of thermal sensation reaches its peak at 30 days, while improvements in mechanical sensory function persist up to 60 days postoperatively [57], potentially due to varying repair rates among different nerve fiber subtypes [58].

Compared to conventional therapies, PBM offers distinct advantages. Randomized data indicate PBM can outperform vitamin B complex in neurosensory recovery [59]; platelet-rich fibrin (PRF) has also shown benefit, although direct head-to-head trials versus PBM are lacking [60]. In a clinical context, the optimization of treatment parameters is essential. Near-infrared wavelengths, specifically within the range of 810–980 nm, are extensively utilized due to their optimal tissue penetration capabilities, in conjunction with energy densities ranging from 7 to 12 J/cm^2^. Initiating therapy immediately following surgery, with sessions conducted 2 to 3 times per week over a period of 3 to 6 weeks, achieves a balance between efficacy and safety [59, 61].

Nonetheless, current research is subject to several limitations. The majority of studies have been characterized by small sample sizes (*n* = 10–30) and short follow-up periods (≤60 days), alongside a lack of standardized protocols regarding treatment parameters such as wavelength, energy density, and irradiation sites [54, 59]. Furthermore, variability in assessment methodologies—such as dependence on subjective VAS scores or inconsistent outcomes from thermal sensory testing—undermines the reliability of the findings.

Despite these challenges, existing evidence supports PBM as a favorable option for postoperative neural management. Future research should focus on multicenter, large-sample trials to establish standardized treatment protocols and to explore the molecular mechanisms involved in the regulation of Schwann cell activation or neurotrophic factor expression, thereby enhancing clinical translation potential [59, 62] (Table 2).

**Table 2:** Photobiomodulation (PBM) therapy for IAN injury management

Category	Technique/strategy	Key research support/technical details	Clinical outcomes/advantages
Mechanisms	Activation of mitochondrial cytochrome c oxidase; anti-inflammatory effects; Schwann cell proliferation	- Enhances ATP synthesis - Stimulates nerve growth factor secretion	- Peak thermal sensation recovery at 30 days [57] - Superior to vitamin B complex [59]
Treatment protocol	Wavelength: 810–980 nm; energy density: 7–12 J/cm^2^; 2–3 sessions/week for 3–6 weeks [59, 61]	- Optimal tissue penetration - Non-invasive and safe	- 68.75% full sensory recovery at 6 months [62] - Reduced pain scores
Limitations	Small sample sizes (*n* = 10–30); lack of standardized protocols	- Subjective assessment variability - Short follow-up periods (≤60 days)	Requires multicenter trials for protocol validation

“Peak thermal recovery at 30 days” is from Ref. [57]; “68.75% full recovery at 6 months” from Ref. [62]; “superior to vitamin B complex” from Ref. [59]; typical sample sizes *n* = 10–30 from Refs. [59–62]; most RCT follow-ups ≤60 days from Refs. [59–61]; commonly used PBM parameters (810–980 nm; 7–12 J/cm^2^; 2–3 sessions/week for 3–6 weeks) from Refs. [59, 61]

#### Corticosteroids in neural management

Corticosteroids are widely used perioperatively to control postoperative inflammation and edema after orthognathic surgery; their effects on neurosensory disturbance (NSD) are agent- and route-dependent, varying with dose, timing, and delivery (e.g., intravenous vs. local administration). In NSD-oriented management, the two corticosteroids most commonly employed are dexamethasone and betamethasone, which therefore form the focus of the following comparison.

Intraoperative local injection of betamethasone into the pterygomandibular space has been shown to significantly enhance postoperative neural recovery. A clinical study conducted by Hamad et al. reported complete restoration of tactile sensation at 6 months postoperatively in the injection group, whereas 31% of the control group, which received sterile distilled water, continued to experience paresthesia [63]. In contrast, direct topical application of dexamethasone to the exposed nerve did not improve neurosensory function, indicating no demonstrable benefit in this context [64].

The preoperative intravenous administration of 40 mg dexamethasone has been shown to effectively reduce postoperative swelling [65], yet it exhibits limited efficacy in mitigating sensory deficits. This observation suggests that the primary mechanism of dexamethasone involves the suppression of inflammatory mediator release and the reduction of capillary permeability, rather than the direct promotion of axonal regeneration. While numerous studies have confirmed the anti-inflammatory properties of dexamethasone [65], its neuroregenerative effects remain inadequate when sustained drug activity at the site of injury cannot be achieved [64].

Dexamethasone demonstrates significant efficacy in reducing acute postoperative inflammatory responses, such as swelling and edema; however, its ability to enhance sensory functional recovery is relatively modest. Local administration has been found to be more effective than systemic delivery. Nonetheless, further investigation is required to determine the optimal dosing (e.g., whether doses exceeding 40 mg are necessary) and timing (intraoperative administration versus postoperative supplementation).

Future directions. Research should pivot to optimizing local betamethasone, the corticosteroid with demonstrated early sensory benefit. Priorities include defining the optimal dose, timing, and injection plane (e.g., pterygomandibular space) and developing sustained-release formulations to maintain perineural exposure while limiting systemic effects. Multicenter, adequately powered trials using standardized quantitative sensory outcomes and ≥6–12-month follow-up are needed to confirm durability. Combination strategies—for example, PBM plus local betamethasone—warrant head-to-head testing against monotherapy. Image-guided delivery could improve precision and reproducibility. By contrast, dexamethasone should presently serve as an edema-control comparator; only novel delivery systems would justify re-evaluation for NSD improvement (Table 3).

**Table 3:** Corticosteroids in neural management post-BSSO

Intervention	Route and dose (timing)	Primary outcome	Clinical notes
Betamethasone (local)	Pterygomandibular-space injection, 6 mg at closure	6-month tactile recovery 100% vs. 69%; early (1–3 months) sensory tests favored treatment [63]	Consider at closure to accelerate early sensory recovery; long-term durability uncertain
Dexamethasone (IV)	Preoperative 40 mg IV, single dose	Edema/swelling reduction; no significant improvement in NSD [65]	Best for acute (≤48 h) inflammation/edema control; not neuroregenerative
Dexamethasone (topical drippage onto exposed IAN)	Intraoperative topical drippage	No NSD benefit [64]	Not recommended for NSD improvement in this setting

Local betamethasone improves early sensory recovery; a study reporting 100% vs. 69% complete recovery at 6 months is Ref. [63]. Preoperative 40 mg IV dexamethasone reduces swelling/pain but shows no significant effect on long-term NSD: Ref. [65]. Intraoperative topical instillation onto the exposed IAN shows no benefit: Ref. [64]. Unless otherwise stated, endpoints refer to clinical sensory testing; imaging endpoints (e.g., CBCT canal penetration) should not be conflated with long-term neurosensory disturbance

While both photobiomodulation (PBM) and corticosteroids offer therapeutic value for IAN injury management, critical differences in evidence quality and clinical intent warrant hierarchical consideration. Corticosteroids present level II evidence (OCEBM 2011): preoperative IV dexamethasone effectively controls acute postoperative inflammation/edema within ≤48 h but has not shown consistent improvement in NSD; direct topical dexamethasone on the exposed IAN is not beneficial; in contrast, local betamethasone (pterygomandibular-space injection) has demonstrated early sensory recovery. PBM provides level I evidence (OCEBM 2011) based on systematic reviews/meta-analyses of randomized trials [54, 62] supporting functional recovery in the subacute phase (>48 h), although parameter standardization remains challenging. Accordingly, the recommendation hierarchy prioritizes: (1) acute phase (≤48 h): IV dexamethasone for edema control, with optional local betamethasone at closure to accelerate early sensory recovery; (2) subacute phase (>48 h): PBM to promote neurosensory recovery; and (3) refractory cases (≥6 months): surgical exploration. This staged approach balances immediacy of anti-inflammatory action with regenerative potential, and Table 4 summarizes the comparative evidence landscape.

**Table 4:** Key differentiators of postoperative interventions

Aspect	PBM	Corticosteroids
Primary action	Nerve regeneration	Anti-inflammatory
Evidence strength	Level I	Level II
Critical time window	>48 h	≤48 h
Long-term benefit	Significant	Minimal
Clinical role	Subacute repair	Acute symptom control

OCEBM 2011 levels of evidence: level 1 = SR/meta-analysis of *RCTs*, level 2 = individual *RCT*, level 3 = nonrandomized cohort/case-control/series. PBM level I based on SR/meta-analyses [54, 62]; corticosteroids level II based on single RCTs supported by cohort studies [63, 65]

#### Methods note (narrative review)

We performed a structured narrative search of PubMed/MEDLINE, Embase, Scopus, Web of Science, the Cochrane Library, and ClinicalTrials.gov (Jan 2000–Sep 2025). Queries combined BSSO/mandibular setback with inferior alveolar nerve (IAN)/neurosensory outcomes and key interventions (fixation: bicortical screws vs. monocortical plates; imaging: CBCT; instruments: piezo/ultrasonic; postoperative: photobiomodulation/low-level laser; corticosteroids: dexamethasone/betamethasone). Inclusion: English, full-text human studies reporting IAN/NSD; selected animal/in vitro data only for mechanism. Exclusion: non–full-text items, editorials, single-case reports, non-BSSO or studies lacking IAN/NSD endpoints. Evidence weighting followed OCEBM (2011): systematic reviews/RCTs > prospective/retrospective cohorts; imaging surrogates (e.g., CBCT canal penetration) were interpreted separately from clinical NSD outcomes.

## Conclusion

**Fig. 1:**
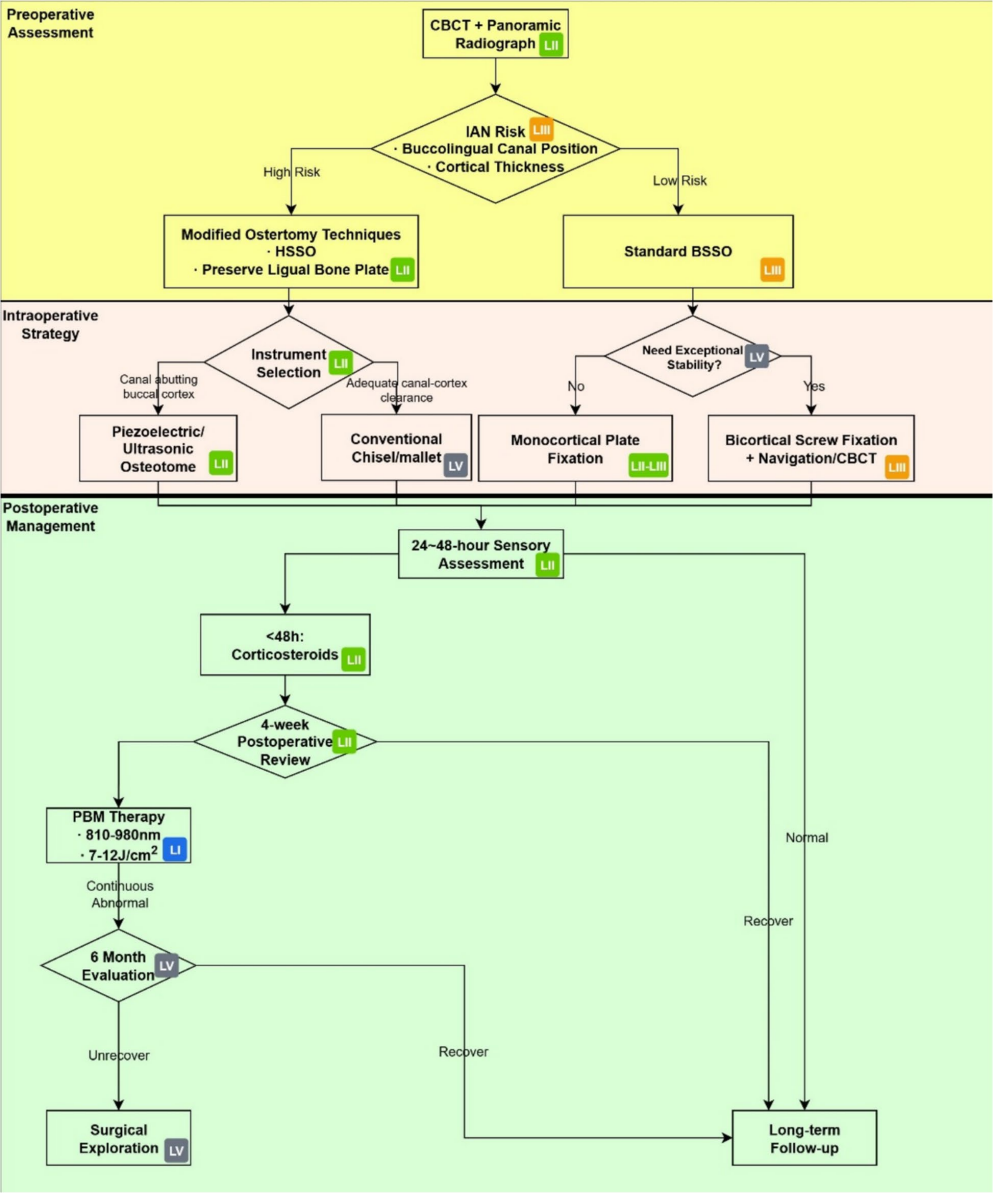
Multimodal clinical pathway for IAN injury prevention and management in BSSO. Abbreviations: BSSO, bilateral sagittal split osteotomy; CBCT, cone-beam computed tomography; HSSO, high oblique sagittal split osteotomy; IAN, inferior alveolar nerve; PBM, photobiomodulation. Evidence grading (OCEBM 2011): level I—systematic reviews/meta-analyses or high-quality randomized trials; level II—lower-quality randomized trials or prospective cohort studies; level III—retrospective cohort or case-control studies; level V—expert opinion/consensus. Ranges such as “level II–III” indicate mixed evidence

Neurosensory disturbance resulting from inferior alveolar nerve injury following bilateral sagittal split osteotomy remains a significant complication that adversely affects patients’ postoperative quality of life. Current research has examined various preventive and therapeutic strategies, including preoperative assessment, intraoperative techniques, and postoperative management. However, existing studies are often limited by inconsistent evaluation criteria and insufficient sample sizes. In addition, imaging surrogates (e.g., CBCT canal penetration) should be differentiated from clinical NSD endpoints when interpreting outcomes. Future research should focus on multicenter collaborative studies to conduct large-scale investigations, establish standardized assessment protocols and treatment guidelines, and elucidate the mechanistic contributions of various factors to IAN injury. Continued advancements in surgical techniques and therapeutic modalities may help reduce the incidence of inferior alveolar nerve injury after bilateral sagittal split osteotomy, improve early recovery, and strengthen the evidence base for clinical decision-making. A multimodal clinical pathway integrating these strategies is proposed in Fig. 1.

## Data Availability

Not applicable.

## Abbreviations

BSSO: Bilateral sagittal split osteotomy
IAN: Inferior alveolar nerve
NSD: Neurosensory disturbance
CBCT: Cone-beam computed tomography
OPG: Panoramic radiography (orthopantomogram)
CT: Computed tomography
BCF: Bicortical screws
MCF: Monocortical miniplates
PBM: Photobiomodulation
NCS: Nerve conduction studies
QST: Quantitative sensory testing
TSEP: Trigeminal somatosensory evoked potentials
